# Experiences of social support by participants with morbid obesity who participate in a rehabilitation program for health-behavior change: a qualitative study

**DOI:** 10.1186/s40795-023-00810-0

**Published:** 2023-12-14

**Authors:** Karoline Thomlevold Jøranli, Linn Tennefoss Vefring, Maria Dalen, Lisa Garnweidner-Holme, Marianne Molin

**Affiliations:** 1 Department of Nursing and Health Promotion, Faculty of Health Sciences, Oslo Metropolitan University, St. Olavs plass, P.O. 4, Oslo, 0130 Norway; 2 Department of Health and Exercise, School of Health Sciences, Kristiania University College, PB1190 Sentrum, Oslo, 0107 Norway

**Keywords:** Health-behavior change program, Social support, Morbid obesity, Group-based intervention, Qualitative

## Abstract

**Background:**

Obesity is a global public health concern with significant implications for individuals’ physical health and overall well-being. Health-behavior change programs are crucial for addressing obesity and its associated health risks. Social support plays a central role in facilitating successful outcomes in these programs, yet limited qualitative research exists on the experiences of individuals with morbid obesity participating in such interventions. Thus, this study explores how participants with morbid obesity experienced social support in a group-based rehabilitation program for health-behavior change.

**Methods:**

Fourteen participants in a group-based rehabilitation health-behavior change program in Norway were interviewed using semi-structured interviews. Data were analyzed with thematic analysis.

**Results:**

The thematic analysis revealed three primary sources of social support: support from other participants in the group, social support from family and friends, and support from the interdisciplinary team. The participants emphasized the significance of ongoing social support throughout their health-behavior change program. Participants appreciated fostering a sense of community and regular interaction with other members of the program to ensure ongoing social support.

**Conclusions:**

Participants outlined the importance of maintaining a sense of community in the group and appreciated platforms for facilitating ongoing interactions and support among group participants. Future studies should focus on long-term interventions, tailored approaches for individuals with diverse needs involving family and friends, and the impact of enhanced peer support. By understanding the role of social support in health-behavior change programs, interventions can be optimized to better support individuals with morbid obesity.

**Supplementary Information:**

The online version contains supplementary material available at 10.1186/s40795-023-00810-0.

## Background

Obesity is a significant global public health concern, and its prevalence continues to rise [1]. In Norway, 24.8% of adult (aged 18 years and over) women and 26.6% of adult men are living with obesity. The prevalence in Norway is slightly lower compared to the European regional average of 25.3% for women but is higher than the regional average of 24.9% for men [2]. Obesity is defined as a body mass index (BMI) greater than or equal to 30 kg/m^2^ and is divided into three subgroups: obesity grade 1 (BMI 30–34 kg/m^2^), obesity grade 2 (BMI 35–39 kg/m^2^), and obesity grade 3 (BMI ≥ 40 kg/m^2^) [3]. The latter grades (2 and 3) are referred to as morbid obesity [4]. Obesity affects morbidity rates, disability, and overall quality of life [5, 6]. Moreover, it is a major risk factor for a range of noncommunicable diseases, including type 2 diabetes, cardiovascular diseases, various types of cancer, osteoarthritis, and other health problems [3]. To address the increasing prevalence of obesity and its associated health risks, it is important to implement effective health-behavior change programs along with preventive measures and interventions at policy level [7, 8].

Several treatment options are available for people with obesity, including health-behavior interventions, pharmacotherapy, bariatric surgery, and combined treatment [3, 4]. Treatment goals of obesity is not only to achieve weight loss but also to enhance the individual’s health and reduce associated health risks [3]. Non-surgical treatment includes dietary change, physical activity, and/or behavioral therapy [7]. In Norway, people with morbid obesity are offered non-surgical treatment and/or bariatric surgery. Non-surgical treatment is usually delivered through a group-based health-behavior intervention program developed and implemented by a team of specialists [9].

A descriptive systematic review showed that while obesity treatments can lead to short-term weight loss, maintaining the weight loss over the long term has proven to be a challenge, with inconsistent results reported [10]. A crucial aspect of all obesity-reducing treatments is permanent health-behavior changes, which should continue throughout the patient’s life to reinforce health-related behavioral changes that aid weight loss [3, 9]. Several studies provide a comprehensive understanding of the challenges and barriers faced by individuals undergoing non-surgical obesity treatment as well as the factors that can contribute to successful outcomes [10–15]. These factors include internal factors (i.e., motivation and self-efficacy), program-specific factors (i.e., the intervention diet), social factors (i.e., supporters and saboteurs), and environmental factors (i.e., an obesogenic environment).

There are promising results for weight-related health behavior programs targeting social support [16]. Social support, involving taking steps to secure or deliver the aid of another person, is identified as an important content of behavior change interventions in Michi et.als’ behavior change technique (BCT) taxonomy [17]. In a review, Tay et al. highlighted the role of social support as a facilitator of weight loss and weight loss maintenance, as it can provide encouragement, motivation, and accountability [14]. Verheijden et al. underscored the importance of social support in health-related behavioral change, defining social support as the availability of potential support-givers (structural support) and the perception of support (functional support) [13]. Their article further explained that social support can include emotional support, practical assistance, advice, guidance, and companionship. Social support can be provided from spouses, family members, friends, co-workers, and healthcare professionals, and it can have a positive impact on an individual’s mental and physical health as well as their overall well-being [13]. Hammarstrøm et al. found that a supportive and motivating group environment was a key facilitator of weight loss, while a lack of social support was a significant barrier to weight loss [15]. Taken together, these studies suggest that group treatment and social support are important components of effective health-behavior change interventions and that interventions should thus aim to promote positive and supportive social environments to facilitate weight loss and weight loss maintenance [13–16].

Through our research, we have found few studies that have specifically investigated the experiences of social support in a health-behavior change program for people with obesity [14, 15]. While there are many quantitative studies on the role of social support in weight reduction, several studies have also acknowledged the scarcity of qualitative research on personal experiences of participating in weight loss interventions [13–15]. According to Hammarström et al., there is a lack of research on the experiences of participants from the general population in dietary interventions, although some qualitative studies have focused on patients with specific diagnoses [15]. In a recent systematic review by Tay et al. [14], the investigators analyzed qualitative data from over 500 participants across different countries between 2011 and 2021. The review identified social support as a crucial facilitator of weight loss and maintenance, both from within and outside the intervention. Additionally, a lack of external support was found to be a significant barrier [14]. By exploring how individuals with obesity perceive social support in initiating and sustaining health-behavior changes, our study seeks to identify areas where future programs can be improved to better support participants in achieving successful outcomes from health-behavior change programs. Specifically, we aimed to explore the experiences of participants with morbid obesity who participated in a group-based rehabilitation program for health-behavior change both in the beginning and during a follow-up period.

## Methods

This study included two interview rounds among participants enrolled in a group-based rehabilitation health-behavior change program at a rehabilitation facility in Norway. The first interview round was conducted among participants during the introduction course. The second interview round was conducted among participants in the follow-up period. The study was conducted in accordance with COREQ guidelines [18].

### Participants

The study included 14 participants, 10 women and four men, with ages ranging from 29 to 58 years. All participants were morbidly obese (BMI ≥ 40 or a BMI ≥ 35 with one or more obesity-related health condition(s)). Participants were referred to a behavior-change program within specialist health services either from their general practitioner or a public hospital. The behavior-change program is part of the public rehabilitation for obese patients from the primary health care services in Norway [19]. The program involved an eight-week introduction course (first round of interviews), where participants took part three times a week. Participants received education and guided group discussions about physical activity, dietary counseling and behavioral change by an interdisciplinary health care team existing of clinical dietitians, psychologists and physiotherapists. The follow-up period lasted up to five years (second round of interviews). In the follow-up period, participants received automatically generated text messages from the program on their mobile every 14 days that encouraged them to send feedback on their progress on physical activity, dietary behavior and weight loss. The main goal of the program is to achieve a 10–15% reduction in participants body weight during the first year. On their own initiative, participants used social media to stay in contact with other group members during the program. Seven participants were about to complete the eight-week introduction course, and seven participants were one to two years into the rehabilitation process. Patients participated without coercion and provided informed written consent.

### Data collection

Data for this study stem from individual interviews about patients’ experiences with a rehabilitation program. MD (MSc in public health nutrition) and MM (professor in public health) developed the semi-structured interview guide guided by interview guides that were used by MM in previous projects among obese patients (Supplementary file 1). The interview guide was pilot-tested among study colleagues of MD as well as participants in previous related projects. The pilot-test lead to minor changes in the wording of questions. MD recruited the participants at the site of the rehabilitation program from October until November 2018. MD presented the study and participants contacted MD if they were interested to participate. Interviews were conducted and transcribed by MD. MD observed an eight-week introductory course to have knowledge about the programs content, in which none of the participants of this study took part. MD did not have any pre-existing relationship with the participants. Interviews lasted 30–60 min (mean 43 min). Interviews were conducted in Norwegian. The interviews were audio-recorded and transcribed verbatim by an external researcher. The recruitment process was conducted until MD had achieved informational power, which means to have high-quality interviews providing sufficient information about the aim of the study [20].

### Data analysis

The analysis focused on the role of social support during participants’ rehabilitation program. The interviews were analyzed thematically using Braun and Clarke’s methodology [21], which involved a systematic approach including the following steps: (i) familiarization, (ii) generating initial codes, (iii) searching for themes, (iv) in-depth review of the themes, (v) defining and naming themes, and (vi) producing the report related to the aim of the study. Analysis was conducted by KJT and LT (both master students in health sciences), and LGH and MM (professors, experienced in qualitative research). A mix of inductive and deductive approach was used to identify the themes [22]: initial codes were generated from participants’ experiences of social support. Since participants clearly distinguished between the sources of social support, codes were arranged into sub-themes and main themes representing the sources of social support. We identified codes in NVivo software (version 12.0). Sub-themes were then identified by grouping similar codes together and identifying themes across the text. The final step in the analysis involved interpreting the identified themes within the context of the research question. As a team, we analyzed each interview before engaging in a mutual comparison and reaching consensus on the coding. To increase the credibility and trustworthiness of our data and subsequent interpretations, any disagreements were resolved by revisiting the original text and engaging in a thorough discussion to ensure the accuracy of the coding. The analysis was conducted in Norwegian. Translation of quotes presented in the research section, sub-themes and main themes was verified for accuracy by a professional language editor.

## Results

The results are presented collectively for both rounds of interviews. The identified themes and subthemes are shown in Table 1.

**Table 1:** Main themes and subthemes

Main themes	General experiences with social support during a health-behavior change program	Social support from other participants in the group	Social support from family and friends	Social support from the interdisciplinary team
Subthemes	Increased feeling of social support	Being in the same situation enhanced the feeling of relatedness	Openness with family and friends	Importance of staying in contact with health professionals
	Increased accountability to oneself and others	A sense of belonging together	Importance of receiving feedback	Appreciated respect and care from the interdisciplinary team
	Importance of regular support	Strengthening motivation and enjoyment related to health-behavior change		
		Staying connected with the group after the program		

### General experiences of social support during a health-behavior change program

#### Increased feeling of social support and accountability

Overall, participants expressed that being part of the program increased their feeling of social support: *“Yes, here I got a lot of support. Both from experts and the group.”* (Participant 5). The participants reported experiencing a sense of responsibility toward their peers to show up, actively participate in meetings, and exercise together. They told to value receiving encouragement and feedback from other participants and explained that this created a positive feedback loop, where they felt motivated by their progress and the support of others and were therefore more likely to continue making positive changes, as illustrated by the following statement by a participant who had just completed the introduction course: “*When you’re being followed up, you’re more focused and sharp. It’s like you’re always trying, always pushing yourself… You don’t want to disappoint yourself or those around you*” (Participant 6).

#### Importance of regular support

Many of the participants expressed concerns about personal motivation with less intensive follow-up, reduced commitment, and implementing the health-behavior changes alone. The participants often highlighted the importance of continued support after the introduction course, illustrated by the following statement by a participant who was over a year into the program and was struggling to maintain new habits: “*[…] I feel that this stay has been a support system or that you are in a position where you get a lot of backup and maybe when you come home to everyday life it’s easy to fall back into old habits*” (Participant 12). A participant from the first group described the challenges of implementing health-behavior changes alone: “*And I live alone, so that’s why I have a little bit of anxiety about not being able to continue with it […] Because then there’s no one to control me anymore, right?*” (Participant 4). The participants stated to highly value regular support provided by the group-based rehabilitation program and the interdisciplinary team, including peer support and guidance from healthcare professionals. Many expressed concerns about maintaining their progress without ongoing support and transitioning back to a different social environment. A participant in the first group expressed this as follows: “*When you don’t have the same level of follow-up as you have here, and you don’t meet every day, then you don’t know how it’s going to be. […] I have received education and that has been a framework, but now I have to try to find other meeting places and try to get into another environment […]*” (Participant 1).

#### Importance of staying connected with the group

The majority of the participants experienced the transition from the introduction course to everyday life as challenging. They told that they often felt left alone without regular guidance and support. The participants emphasized the challenge of transitioning from the close social support provided by the group-based rehabilitation program and the interdisciplinary team to a setting with less regular guidance and support. One participant from the second group explained the difficulties in the following way: “*But then you meet life without necessarily having someone to come to every other day as we did at first, and then eventually all of these things you struggle with, call them ghosts, all the things that have made you who you are in terms of weight and health-behavior, they come back with full force, without knowing that in two days you’re going back there, and you’ll get support. So, there was a difference*” (Participant 9). In this subtheme, there was a notable discrepancy between the two interview groups. The participants who had recently completed the introduction course expressed concerns and fears about transitioning to an unstructured daily routine, while those who had already experienced the transition talked about the challenges that arose and their feelings regarding less regular guidance and support after the introduction course. Of the latter, several participants found the change from a structured program to an unstructured routine overwhelming and felt that additional resources and strategies were needed to maintain the progress they had achieved.

### Social support by other participants in the group

#### Being in the same situation enhanced relatedness

Many of the participants stated that being in the same situation with other program members enhanced their feeling of relatedness. One participant from the first group illustrated this as follows: “*[…] Even though there are very big differences between us as well, both in terms of size, challenges and health issues there are also similarities […] I would almost say that having people around you who have exactly the same challenges, or at least many of the same challenges, is the most important thing*” (Participant 3). Participants experienced the feeling of being with like-minded peers, despite being different, as positive and that it helped to establish a supportive and encouraging atmosphere. A participant from the second group explained the relatedness in this way: “*We can get in touch with each other and ask, ‘How are you doing?’ And tell them, ‘Actually, things have been a bit tough for me lately. Can you give me a little boost?’ And that’s really great because we are all in the same situation*” (Participant 10).

#### Sense of belonging together

The participants said that they appreciated emotional support from peers in the group and perceived that this support promoted a sense of belonging and social connection. One participant stated: “*[…] I think the biggest benefit has been that we can talk to each other. Many have dared to open up and as a result, we have gotten more people to open up*” (Participant 2). They said that the group provided a supportive environment in which they felt safe to exchange tips and receive encouragement from their peers.

#### Strengthening motivation and enjoyment related to health-behavior change

They also expressed that being part of group activities enhanced motivation and enjoyment related to health-behavior change. One participant described how the group contributed to motivation and support: “*We’ve motivated each other, we have an incredibly good group, and you get caught up, you really do […] you get support from the group*” (Participant 2). Another participant explained that training in a group leads to a desire to push harder: “*[…] When you have people around you, and you perform in a group it’s like you give a little extra. […] And you push boundaries*” (Participant 6). The participants emphasized the social aspect of group training and explained how it created a sense of belonging and provided opportunities for further social connections.

Staying connected with the group after the program was perceived as useful for providing a sense of security and support. Participant 2 told: *“We have our own Facebook group that helps us to stay connected with each other.*” Participants mentioned that social media played a crucial role in fostering social support by another group member during the program. They described how they used various platforms, such as Snapchat and Facebook, to stay connected, praise each other, and provide support when completing a training session or reaching a goal. Nevertheless, some participants also mentioned that it could be mentally demanding to deal with a Facebook group during the process of health-behavior change. These participants found it more manageable to communicate and stay in touch with certain individuals rather than the entire group.

### Social support from family and friends

#### Openness with family and friends

Most of the participants highlighted that being open with family and friends contributed to their experience of social support in the health-behavior change process. They emphasized the importance of family members who implemented changes and actively participated in the process alongside them. One participant expressed the great value of support: “*Yes, it has been crucial to have a husband who has been very supportive, always supportive in a way, and he was overweight himself*” (Participant 8). Another participant reported receiving natural support at home and how this contributed to making the health-behavior change easier by having generally healthy eating habits. The participants explained that if people in their social network were aware of their health-behavior change, it could contribute to and increase their feeling of being supported because they helped to promote and facilitate their healthy health-behavior choices. Being open with friends and family could help the participants feel less alone during the process of health-behavior change, as demonstrated by the following statement: “*It helps to be open with friends about the changes you are going through during the program, but also afterwards, so that they support you.”* (Participant 6). One participant explained how openness about participating in a health-behavior change program contributed to friends becoming a greater source of support, with some even joining in on the changes and providing motivation: “*And I think that by aligning with others and being a bit open about this, others can help you reach that goal*” (Participant 8). In addition, the participants indicated that being open about their process in the workplace could lead to support and understanding from their supervisors and other colleagues. One participant described this experience: “*Uh, my boss at work has been pretty supportive and asked if everything is going okay and sometimes tried to have healthy food at evening meetings and stuff*” (Participant 11). Conversely, some participants said a lack of support from their families was a difficulty they encountered.

#### Importance of receiving feedback

The participants highlighted the importance of receiving support and feedback from their family and friends, as it helped them recognize progress that they may have overlooked. They found such validation from an outsider’s perspective to be especially valuable when they did not feel the change themselves, for example, through comments like “*Now we think you look great*” or “*Wow, you’ve lost weight*” (Participant 10). The participants stated that such encouraging comments regarding their health-behavior change process from friends and family increased their feeling of being supported, which helped increase their motivation and made it easier to maintain the changes and good habits. However, opinions and comments from friends and family could also be challenging, such as “*You must not be so hard on yourself or so strict with yourself, you must not condemn yourself for it*” (Participant 4).

### Social support from the interdisciplinary team

#### Importance of staying in contact with health professionals

The participants appreciated the opportunity to contact the interdisciplinary team and perceived this as supportive of their health-behavior change process, as exemplified by participant 7: “*They [health professionals at the rehabilitation program] told me that they are here for me, and I think that I will come back to this opportunity. So, I think that they can support me because I really want to succeed*.”. The participants appeared to value individual conversations with members of the interdisciplinary team and stated that it made them feel seen as individuals and not just as part of a larger group. Further, they appreciated the availability of the interdisciplinary team and the opportunity to contact a professional if they needed help or guidance as essential for their progress. One participant from the second group said the interdisciplinary team’s support was vital in their health-behavior change process: *“…So it’s really nice to know that you can just contact a nutritionist or psychologist or whoever it may be, to get back on track again. I’m still in a process where things aren’t going as smoothly as I’d like for my own sake […] It’s like getting a refresher on what you’ve learned before. With a little bit more input regularly, it’s easier to stay focused on it*” (Participant 10). According to the participants, knowing that the team was available gave them a sense of security and support and made them feel comfortable seeking help if they needed it.

#### Appreciated respect and care

Respect and caring from the interdisciplinary team fostered a sense of trust and security, as explained by participant 3: “*Those who work here really care about us. They treat us with respect […] I have never been to a place where they take us so seriously*.” Several participants credited their achievements in health-behavior change to the interdisciplinary team’s exceptional support. One participant expressed a deep sense of pride in their progress, which they attributed to the nutritionist’s enthusiastic and charismatic approach. Another participant emphasized the importance of connecting with the psychologist, stating that it was essential to the overall success of the health-behavior change process: “*[…] And the sessions with the psychologist have been (absolutely) crucial for me and it’s important to emphasize that*” (Participant 8). The participants also highlighted the need for social support through personalized and individual follow-up after the introduction course. In particular, one participant emphasized the need for different types of support based on each individual’s needs: “*Because I see in my group now, we struggle with different things, even though some of it is similar […] some may need a nutritionist, while others may need more psychological support […]. So I wish there had been a little more follow-up after the eight weeks, not just that you can come and weigh yourself here if you want*” (Participant 9).

## Discussion

The findings of this study are consistent with the existing literature on the role of social support in health-behavior change programs [10–14]. The participants in our study reported that being part of a health-behavior change program increased their feeling of social support, which in turn influenced their motivation, commitment, and overall success in making positive changes. Compared to these other studies [6–10], our study participants were at different stages of the behavior-change program. All of our participants appreciated social support from the group during the program. In particular, participants interviewed during the introduction course were afraid of losing this support after the program. Also, participants in the follow-up period were concerned about the change from a structured program to unstructured routines. They felt the need for additional resources and strategies to maintain their achieved progress.

An increased understanding of predictors of successful weight loss amongst individuals with obesity is important to develop effective health-behavior programs. According to a systematic review, the majority of high-quality follow-up treatment studies of individuals with obesity are not successful in maintaining weight loss over time [10]. A qualitative study among people with obesity and other stakeholders identified social support as an important BCT for long-term weight loss in eHealth interventions [23]. Also, Elfhag et al. identified social support as an important factor for weight maintenance [12]. Our analysis of the interviews indicates a need for social support throughout the entire process of health-behavior change. However, the participants’ sources of support changed noticeably over time. The participants who had recently completed the introduction course highlighted the significance of both the group and the interdisciplinary team as important sources of social support, whereas those who had been in the program for one to two years placed less emphasis on the group and more on the interdisciplinary team. By providing regular opportunities for interaction, interventions can foster a sense of community and ensure ongoing social support throughout the entirety of the program [14]. Swancutt et al. highlighted the role of healthcare professionals in facilitating and managing the group process in ways that encourage patients to form meaningful psychological connections with each other and a shared social identity [7].

### Social support from other participants in the group

Within the group, the participants highlighted the benefits of shared experiences and the feeling of relatedness that emerged from being in a similar situation. This finding is in line with the SCT, outlining that people learn from others in a social context [24]. The group enhanced their feeling of social support, providing encouragement, motivation, and a sense of belonging. A review by Swancutt et al. [7] on group-based interventions for people with severe obesity supports our findings regarding the importance of group social support. Their review highlights the potential advantages of group settings, such as peer support, shared social identity, and a sense of belonging, and points to how this can enhance motivation and adherence to health-behavior changes [7]. These findings are further supported by an international systematic review from New Zealand and a conceptual review from Sweden [12–14], both of which emphasize the importance of social connection and emotional support. The group activities and training sessions further strengthened the participants’ enjoyment and motivation to make positive changes. The positive influence of group-based interventions found in the current study is in line with the findings of Tay et al., who demonstrated the advantages of integrating a group component into an intervention, as it inspires participants, with a sense of community, and for some, a competitive environment [14]. In our study, participants described how they stayed connected with others in the group through social media. Even though there is an increasing body of the literature investigating the effectiveness of social-media-based weight-loss programs [25], our study demonstrates that social media might be a useful tool to enhance social support during an in-person weight-loss programs. However, it has to be acknowledged, that some of our participants preferred to have individual contact with other members of the program, prior to participating in the digital group.

### Social support by family and friends

The participants emphasized the importance of social support from family and friends. They reported that having supportive family members who actively participated in the health-behavior change process alongside them was particularly valuable. This finding is similar to a qualitative interview study from Denmark on important drivers of long-term personal health-behavior changes from a patient perspective, in which one of the main identified themes was support from family and peers [26] as well as the study of Verheijden et al., which highlighted the role of social support from family members in health-behavior-focused weight management interventions [13].

Some participants mentioned challenges related to family members who did not support their health-behavior changes. This made it harder for them to implement and maintain healthy habits and health-behavior changes. The influence of friends and family on weight loss efforts can be both positive and negative; whereas some friends and family members helped the participants maintain healthy eating habits, others acted as saboteurs [14, 27]. These findings underscore the significance of incorporating social support from family and friends into weight management interventions as well as the need to address both the positive and negative aspects of social interactions to support individuals’ efforts to maintain healthy habits [12, 14, 15].

### Social support from the interdisciplinary team

The findings from this study align with previous research on the importance of social support provided by an interdisciplinary team in the health-behavior change process [10, 13, 14]. The participants in our study emphasized the value of being able to contact the interdisciplinary team and to seek guidance and help when needed. The participants greatly appreciated the availability of the interdisciplinary team, as it gave them a sense of security and comfort. Tay et al. found that participants placed significant value on personalized support and accountability, which not only fostered trust in healthcare professionals but also played a crucial role in facilitating successful outcomes [14]. Further, our analysis revealed that the participants expressed concerns and fears regarding the transition from the structured health-behavior change program to everyday life. They emphasized the difficulties of sustaining progress without regular guidance and support from the interdisciplinary team. According to Tay et al., the discontinuation of supervision after an intervention can be a significant barrier to weight loss maintenance, causing the participants to feel unsupported and lacking guidance, thus leading to a sense of uncertainty and difficulty in sustaining their progress [14]. As described in the introduction, Verheijden et al. distinguish between structural and functional social support [13]. Structural support refers to the availability of potential support-givers, while functional support refers to the perception of support. Our participants appreciate structural support, through other participants, family and friends and health professionals. However, functional support shows a stronger correlation with health [13].

This study has some limitations. The findings of this study are not generalizable. However, they can be transferable to patients participating in comparable health-behavior interventions for weight loss. Transcripts were not returned to participants for comments. However, three investigators listened to the audiotapes and secured the accuracy of the transcription. Our results could have been strengthened by interviewing the same sample over time instead of comparing two different groups at different stages in the rehabilitation program.

## Conclusions

In conclusion, the participants in this study outline the importance of social support in health-behavior change programs. Recognizing various sources of support and implementing strategies that foster relatedness and emotional support were identified as key factors in the social support experienced by the participants. Recommendations to enhance social support in lifestyle-change programs are to provide platforms for participants to facilitate interactions among group members and their health professionals during and after the program. Future studies should focus on examining long-term health-behavior interventions, developing tailored approaches to meet the diverse needs of individuals with morbid obesity, exploring effective strategies for involving family and friends in these programs, and examining the impact of enhanced peer support.

### Electronic supplementary material

Below is the link to the electronic supplementary material.

Supplementary Material 1

## Data Availability

Available on request.
