# Supplementary material for: Experiences of social support by participants with morbid obesity who participate in a rehabilitation program for health-behavior change: a qualitative study
Source: BMC Nutr. 2023 Dec 14;9:149. doi: 10.1186/s40795-023-00810-0 (PMC10722673; doi:10.1186/s40795-023-00810-0)
Supplement: Supplementary file 1 — Supplementary Material 1 [file 40795_2023_810_MOESM1_ESM.docx]

Interview guide

Focus 1: Expectations for the rehabilitation program

*What do you expect from the program?*

Focus 2: Motivation for behavior change

*Why do you want to participate in the program?*

Focus 3: Experiences of social support during behavior-change

*Can you tell me about your need and experiences with support for behavior-change?*

Focus 4: Experience of social support during the behavior-change program

*Can you tell me about your need and experiences with support during the program?*

Focus 5: Suggestions to improve the behavior-change program to enhance social support

*Can you describe what fostered social support during the program?*
